# Severe liver disease related to chronic hepatitis C virus infection in treatment-naive patients: epidemiological characteristics and associated factors at first expert centre visit, France, 2000 to 2007 and 2010 to 2014

**DOI:** 10.2807/1560-7917.ES.2017.22.30.30582

**Published:** 2017-07-27

**Authors:** Alice Sanna, Yann Le Strat, Françoise Roudot-Thoraval, Sylvie Deuffic Burban, Patrizia Carrieri, Elisabeth Delarocque-Astagneau, Christine Larsen

**Affiliations:** 1 Santé publique France, French National Public Health Agency, Saint-Maurice, France; 2 Hôpital Henri Mondor, Assistance Publique-Hôpitaux de Paris, Université Paris-Est Créteil, Paris, France; 3 IAME (Infection Antimicrobials Modelling Evolution), UMR1137 INSERM, Université Paris Diderot – Sorbonne Paris Cité, Paris, France; 4 LIRIC (Lille Inflammation Research International Center), UMR995 INSERM, Université de Lille, CHRU de Lille, Lille, France; 5 SESSTIM (Sciences Economiques & Sociales de la Santé & Traitement de l’Information Médicale), UMR912 INSERM, Aix-Marseille Université, IRD, Marseille, France; 6 ORS PACA (Observatoire Régional de la Santé Provence Alpes Côte d’Azur), Marseille, France; 7 B2PHI (Biostatistics, Biomathematics, Pharmacoepidemiology, and Infectious Diseases), UMR 1181 INSERM, Université de Versailles Saint-Quentin-en-Yvelines, Institut Pasteur, Paris, France

**Keywords:** hepatitis C, surveillance, severe liver disease, public health policy, screening, direct-acting antiviral drugs, injecting drug users

## Abstract

Given recent profound improvements in the effectiveness of antiviral treatment for chronic Hepatitis C virus (HCV) infection, we aimed to describe the characteristics of patients referred to hepatology expert centres in France from 2000 to 2007 and from 2010 to 2014, and to identify factors associated with severe liver disease at their first visit for evaluation. We analysed data from two sources covering all of France: the former hepatitis C surveillance network, which included patients between 2000 and 2007, and the ANRS CO22 HEPATHER multi-centre cohort, which included patients between 2012 and 2014. Severe liver disease (SLD) was defined as the presence of either cirrhosis (histological, biochemical or clinical) or hepatocellular carcinoma. Multivariable Poisson regression models were used to identify the factors associated with SLD in complete-case analysis and after multiple imputation. Overall, 16,851 patients were included in the analysis and SLD was diagnosed in 11.6%. SLD at first visit was significantly associated with known risk factors (male sex, history of excessive alcohol intake, HCV genotype 3), late referral to hepatologists after diagnosis and HCV diagnosis at an older age. Providing earlier specialised care and treatment may be an important target for public health action.

## Introduction

French public health policies have targeted hepatitis C virus (HCV) infection since the mid-1990s. Health authorities have promoted HCV-screening among individuals at risk of infection, and have enhanced and improved access to specialised care and antiviral treatment. They have also effectively reduced HCV transmission in the following contexts: blood transfusion [1], healthcare and PWID (people who inject drugs) [2].

France is a low endemic country for HCV infection. In 2004, the prevalence of chronic HCV infection in the general population was estimated at 0.53% (95% confidence interval (CI): 0.40–0.70), corresponding to 232,196 adults (95% CI: 167,869–296,523) 18–80 years of age, nearly 43% of whom were unaware of their infection [3]. Among the infected PWID population, 91% were aware of their infection, but among the blood transfusion recipients, only 50.7% were [3]. Prevalence has tended to decrease since then, with prevalence in 2011 being 0.42% (192,700 adults) [4]. In addition, the estimated number of undiagnosed chronically HCV-infected individuals has also decreased (72,102 adults in 2014) [5].

Chronic HCV infection can evolve into cirrhosis in 10–20% of cases over a period of 20 to 30 years. Cirrhosis is its main complication, along with hepatocellular carcinoma (HCC) [6]. Alcohol abuse, hepatitis B virus (HBV) and human immunodeficiency virus (HIV) co-infections, as well as metabolic disorders have all been shown to be major determinants of liver disease progression to cirrhosis, but individual-specific variations exist because of virus-host interactions. Cirrhosis and HCC have a significant impact on morbidity and mortality related to chronic HCV infection [6]. However, successful treatment of the viral infection can stop evolution to severe liver disease (SLD), can limit the risk of cirrhosis decompensation and its associated liver-related mortality [7] and can even lead to fibrosis regression [8].

Results from treatment with new direct-acting antivirals (DAAs) show very high success rates (sustained virologic response (SVR) rates can reach up to 95–99% with slight variations according to the viral genotype), good tolerance and short therapeutic course [9].

In most European countries, access to these drugs is limited because of their high cost. Priority is given to patients with significant liver fibrosis or cirrhosis (METAVIR score F2-F4), extra-hepatic complications, or HBV or HIV co-infection [10]. However, to increase treatment effectiveness, treatment before the occurrence of cirrhosis (i.e. before METAVIR score F4) is preferable. In fact, SLD is an important negative predictor of SVR in DAAs-based therapy. Moreover, SVR in patients with a METAVIR score of F4 do not necessarily protect from further hepatic complications (e.g. decompensated cirrhosis, HCC) [9].

In France, hepatology expert centres have a pivotal role in chronic HCV infection evaluation and treatment. Until June 2016, their approval was necessary for any HCV antiviral drugs prescription [11].

Given these points, a better understanding of factors associated with late-stage liver disease in patients seeking care at an expert centre for the first time would help inform public health policymaking. It would indeed allow patients identified as at risk of developing hepatic complications to benefit from closer follow-up, and earlier referral and treatment access. Concrete interventions could include increasing HCV screening coverage, comorbidity prevention, and training of physicians involved in the follow-up of such patients.

The main objective of our study is to describe the chronic HCV-infected population seeking care at the hepatology expert centres in France from 2000 to 2007 and from 2010 to 2014, and to identify the factors associated with having HCV infection-related SLD at the time of their first evaluation there.

## Methods

### Study population

For the present analysis, we included data from patients who sought care for chronic HCV infection at hepatology expert centres across France from two periods: 2000 to 2007 and 2010 to 2014.

#### Patients with first visit to an expert centre in 2000–2007

For the period 2000 to 2007, patients were recruited by the hepatitis C hospital service-based surveillance network coordinated by Santé publique France (the French National Public Health Agency) [11]. A total of 26 of the 30 hepatology expert centres located in university hospitals throughout France participated in the network. Every newly-referred adult (≥ 18 years of age) patient with anti-HCV antibodies visiting any of these 26 centres (as an outpatient or inpatient) for the first time was included after consent and without further inclusion criteria.

#### Patients with first visit to an expert centre in 2010–2014

For the period 2010 to 2014, patients who agreed to participate in the nationwide multi-centre cohort study ANRS (France Recherche Nord & Sud Sida-HIV Hépatites) CO22 HEPATHER (ClinicalTrials.gov, number: NCT01953458) [12] that actively recruited individuals infected with HBV or HCV in 2012–2014, were also included. A total of 32 expert centres were involved in cohort recruitment, 26 of which had participated in the former hepatitis C surveillance network. Every adult (≥ 18 years of age) attending centres for HCV or HBV infection follow-up in 2012–2014 was eligible for inclusion in the cohort, regardless of infection duration and duration of the follow-up at the expert centre, with the exception of HIV co-infected patients, pregnant women and adults who could not independently provide consent to participate.

For our analyses, data for patients from these two populations that met the following criteria were included: we selected individuals (i) seeking care for chronic hepatitis C as defined in the following section, (ii) with no history of liver biopsy at the time of first expert centre visit, (iii) who were antiviral treatment naive, and (iv) whose first expert centre visit occurred in the 24 months preceding inclusion in the ANRS CO22 HEPATHER cohort study so as to avoid overlap between the two study periods. Analyses were also restricted to patients who (v) were HIV-negative and (vi) 18 years of age or older.

### Data collection

For all patients in the study, standardised forms were used to collect data from the visit (interview and patient assessment) on the following: sociodemographic characteristics; history of HCV infection; HCV infection risk factors; stage of liver disease when first examined at expert centre (liver fibrosis was evaluated by METAVIR score, clinical signs and liver biochemical markers such as alanine aminotransferase (ALT)); comorbidities such as alcohol consumption and HBV–co-infection; HCV-RNA viral load; and HCV genotype. We analysed data collected at the time of patients’ first visit to the centres or, when this was not possible, the earliest available data from a consultation after the first visit.

### Definitions

Chronic hepatitis C was defined as testing positive for anti-HCV antibodies with persistent detection of HCV-RNA for at least six months after diagnosis.

Liver fibrosis was assessed either by invasive (liver biopsy) or validated non-invasive methods based on serum biomarkers (FibroTest) or based on liver stiffness measurement by transient elastography (FibroScan) [13]. Liver fibrosis assessments were considered for the present analysis if they were performed not more than 12 months before or after the first visit to an expert centre.

In the absence of liver fibrosis assessment, cirrhosis was assessed by a clinical evaluation, which included a physical examination, biochemical tests and imaging, mainly abdominal ultrasound (Table 1). Clinical evaluation was taken into account if performed at the time of first expert centre visit or in the 24 months before inclusion in the ANRS CO22 HEPATHER cohort.

**Table 1:** Study criteria for presence of cirrhosis, France, 2000–2007 and 2010–2014

Liver fibrosis assessment	Criteria for cirrhosis
**Yes**	**Liver biopsy:** METAVIR score F4
	**Serum biomarker (in absence of liver biopsy):** FibroTest ≥ 0.75 [34]
	**Transient elastography (in absence of liver biopsy and serum biomarkers):** FibroScan liver stiffness ≥ 12.5 kPa (cut-off correlated with METAVIR score F4) [35]
**No**	**Clinical evidence of cirrhosis:** association of clinical signs, laboratory findings and imaging [36].

SLD was defined by the diagnosis of either cirrhosis or HCC.

Excessive alcohol intake was defined as more than 140 g of pure ethanol per week for women and more than 210 g per week for men, corresponding to 14 and 21 glasses of wine, respectively. Both present and past consumption were recorded at the time of the interview.

Endemicity of HCV infection in countries of birth was defined according to anti-HCV prevalence estimated by Gower et al. [14] and Lavanchy et al. [15]. Global HCV prevalence data were then categorised in five quintiles: ≤ 0.85%, 0.86–1.4% and 1.5–2% (low HCV prevalence), 2.1–3.2% (intermediate HCV prevalence), and > 3.2% (high HCV prevalence).

Patients’ referral years were categorised into three periods: 2000 to 2003, 2004 to 2007 and 2010 to 2014.

The ALT ratio was calculated as function of the upper limit of normal; during the first two periods, this data was already reported as a ratio and during the last period, it was calculated using an upper limit of normal compatible with that used by clinicians during the first period (35 IU/L).

### Statistical methods

Patient characteristics were presented as numbers and proportions for each category of qualitative data, and as medians and interquartile ranges (IQR) for quantitative data. For each variable, the number and proportion of missing data were also reported.

Bivariate analyses according to the stage of the liver disease (SLD or not) were performed using Poisson regression models. Bivariate modelling of explanatory variables was performed by introducing fractional polynomials to find the best relationship between risk of SLD and the independent variable [16].

Missing data were considered as resulting from a missing at random (MAR) mechanism. A multiple imputation was performed using chained equation [17]. We included all the variables from the multivariate model in the imputation model to ensure that both models (for imputation and analyses) were congenial [17]. For each non-Gaussian continuous variable, we applied the transformation proposed by Nevalainen et al. [18]. We generated 100 imputed datasets. The distributions before and after imputation were compared for every selected variable and were found to be similar.

Factors associated with SLD were identified using multivariate Poisson regression [19] with robust variance and fractional polynomials for continuous explanatory variables [16,20]. The following co-variables were identified a priori and were included in the multivariable model if they had a p value < 0.20 in the bivariate analyses: sex, country of birth, age at HCV diagnosis, circumstances of HCV diagnosis, time between HCV diagnosis and first expert centre visit, French area/region of first expert centre visit, study period at time of first expert centre visit, HCV infection risk factor, excessive alcohol intake, HCV genotype, HBV co-infection and ALT ratio. They were selected using a manual stepwise backward approach. The area/region of referral was forced into the model as an adjustment variable, in order to partially take into account a potential centre effect. We tested interactions between sex and risk factors, sex and excessive alcohol intake, and risk factors and excessive alcohol intake. We also tested interactions between the study period and, respectively, excessive alcohol consumption, HCV infection risk factors, circumstances of diagnosis and HCV genotype. These analyses were performed on both complete cases and after imputation to estimate adjusted prevalence ratios (aPR), their 95% confidence intervals (95% CI) and p values.

All the analyses were performed with STATA 12 (StataCorp, College Station, Texas, United States) and R (version 3.2.3) statistical software. All tests were considered significant with a two-tailed p value ≤ 0.05. The Stata user-written programme ICE was used to perform the imputation process.

### Ethical statements

Protocols for the two studies were approved by the French data protection authority (CNIL) and explained to all patients meeting the case definition, and who provided written consent when enrolled.

## Results

A total of 16,851 patients matched our inclusion criteria: 15,529 from the hepatitis C surveillance network and 1,322 from the ANRS CO22 HEPATHER study (**Table 2**). Of these patients, 55.7% were men, 72.5% were born in low-endemic areas for HCV infection (HCV infection prevalence ≤ 2%), mostly France and other European countries, and 8.1% were born in areas with high HCV prevalence. The proportion of patients born in areas with high HCV prevalence varied from 6.1% in 2000–2003 to 15% in 2010–2014. The median age at diagnosis was 44 years (IQR: 35-56), corresponding to a median age of 43, 44 and 49 years in the three study periods, respectively.

**Table 2:** Characteristics of patients with chronic hepatitis C at time of their first visit to a hepatology expert centre, France, 2000–2007 and 2010–2014 (n = 16,851).

Patient characteristics		Overall		Period of first visit at expert centre following referral					
				2000–2003^a^		2004–2007^a^		2010–2014^a^	
		n or (median)	% or (IQR)	n or (median)	% or (IQR)	n or (median)	% or (IQR)	n or (median)	% or (IQR)
**Total number of patients**		16,851	NA	8,648	NA	6,881	NA	1,322	NA
**Sex**	**Female**	7,374	43.8	3,848	44.5%	3,011	43.8	515	39
	**Male**	9,386	55.7	4,709	54.5	3,870	56.2	807	61
	**Missing**	91	0.5	91	1.1	0	0	0	0
**Country of birth**	**France**	10,615	63	5,762	66.6	3,988	58	865	65.4
	**Europe (outside France)**	818	4.9	336	3.9	380	5.5	102	7.7
	**North Africa and Middle East**	973	5.8	430	5	426	6.2	117	8.9
	**Sub-Saharan Africa**	802	4.8	327	3.8	330	4.8	145	11
	**Asia, Pacific, Americas**	622	3.7	229	2.6	304	4.4	89	6.7
	**Missing**	3,021	17.9	1,564	18.1	1,453	21.1	4	0.3
**HCV endemicity in country of birth** ^b^	**≤ 0.85%**	10,722	63.6	5,812	67.2	4,029	58.6	881	66.6
	**0.86–1.4%**	738	4.4	318	3.7	295	4.3	125	9.5
	**1.5–2%**	751	4.5	364	4.2	299	4.3	88	6.7
	**2.1–3.2%**	219	1.3	64	0.7	129	1.9	26	2
	**> 3.2%**	1,363	8.1	526	6.1	639	9.3	198	15
	**Missing**	3,058	18.1	1,564	18.1	1,490	21.7	4	0.3
**Age at HCV infection diagnosis**	**Years**	(44)	(35–56)	(43)	(34–56)	(44)	(35–55)	(49)	(40–57)
**Circumstances of HCV infection diagnosis**	**Systematic screening**	8,344	49.5	3,911	45.2	3,466	50.4	967	73.1
	**Exposure to a HCV infection risk**	3,145	18.7	1,871	21.6	1,182	17.2	92	7
	**Symptoms or laboratory findings**	3,551	21.1	2,037	23.6	1,292	18.8	222	16.8
	**Unknown**	1,811	10.7	829	9.6	941	13.7	41	3.1
**Time between HCV diagnosis and first expert centre visit**	**Months**	(4)	(2–32)	(4)	(1–27)	(5)	(2–39)	(5)	(2–38)
**French area/region of first visit**	**Paris area**	3,481	20.7	1,775	20.5	1,259	18.3	447	33.8
	**North-West**	3,825	22.7	2,200	25.4	1,504	21.9	121	9.2
	**North-East**	2,978	17.7	1,545	17.9	1,184	17.2	249	18.8
	**South-West**	2,586	15.3	1,370	15.8	1,039	15.1	177	13.4
	**South-East**	3,746	22.2	1,644	19	1,788	26	314	23.8
	**French Caribbean islands**	235	1.4	114	1.3	107	1.6	14	1.1
**HCV infection risk factor**	**Intravenous drug use**	5,234	31.1	2,756	31.9	2,105	30.6	373	28.2
	**Nasal drug use**	347	2.1	151	1.7	139	2	57	4.3
	**Blood-derived product transfusion ** **before 1991**	4,402	26.1	2,427	28.1	1,679	24.4	296	22.4
	**Other risk factors**	4,029	23.9	1,863	21.5	1,574	22.9	592	44.8
	**No risk factor found**	2,839	16.8	1,451	16.8	1,384	20.1	4	0.3
**HBV co-infection at first visit**	**No**	12,283	72.9	5,764	66.7	5,523	80.3	996	75.3
	**Yes**	315	1.9	149	1.7	141	2	25	1.9
	**Missing**	4,253	25.2	2,735	31.6	1,217	17.7	301	22.8
**Excessive alcohol intake** ^c^	**None**	10,946	65	5,459	63.1	4,742	68.9	745	56.4
	**Current**	466	2.8	288	3.3	178	2.6	0	0
	**Current and past**	1,293	7.7	815	9.4	426	6.2	52	3.9
	**Past**	2,303	13.7	1,049	12.1	910	13.2	344	26
	**Missing**	1,843	10.9	1,037	12	625	9.1	181	13.7
**HCV genotype**	**1**	7,023	41.7	3,198	37	3,142	45.7	683	51.7
	**2**	1,391	8.3	683	7.9	601	8.7	107	8.1
	**3**	2,402	14.3	1,125	13	1,065	15.5	212	16
	**4**	1,146	6.8	471	5.4	511	7.4	164	12.4
	**5, 6 or 7**	281	1.7	132	1.5	118	1.7	31	2.3
	**Missing**	4,608	27.3	3,039	35.1	1,444	21	125	9.5
**ALT ratio** ^d^	**Times the upper limit of normal**	(1.5)	(1–2.5)	(1.5)	(1–2.5)	(1.5)	(1–2.3)	(1.71)	(1.03–2.91)
**Severe liver disease^e^**	**None**	13,566	80.5	7,053	81.6	5,622	81.7	891	67.4
	**Cirrhosis**	1,798	10.7	710	8.2	748	10.9	340	25.7
	**Hepatocellular carcinoma**	151	0.9	57	0.7	66	1	28	2.1
	**Missing**	1,336	7.9	828	9.6	445	6.5	63	4.8
**Severe liver disease diagnostic tool**	**No severe liver disease**	13,566	80.5	7,053	81.6	5,622	81.7	891	67.4
	**Liver biopsy**	834	4.9	485	5.6	283	4.1	66	5.0
	**FibroTest**	391	2.3	0	NA	301	4.4	90	6.8
	**FibroScan**	107	0.6	0	NA	0	NA	107	8.1
	**Clinical evaluation**	617	3.7	282	3.3	230	3.3	105	7.9
	**Missing**	1,336	7.9	828	9.6	445	6.5	63	4.8

ALT: alanine aminotransferase; HCV: hepatitis C virus; IQR: interquartile range; NA: not applicable.

^a^ Data for the time periods 2000–2003 and 2004–2007 came from France’s hepatitis C surveillance network while that for the time period 2010–2014 came from the nationwide multi-centre cohort study ANRS CO22 HEPATHER.

^b^ Endemicity of HCV in countries of birth was defined according to anti-HCV prevalence estimated by Gower et al. [14] and Lavanchy et al. [15]. Global HCV prevalence data were then categorised into 5 quintiles: ≤ 0.85%, 0.86–1.4% and 1.5–2% (low HCV prevalence), 2.1–3.2% (intermediate HCV prevalence) and > 3.2% (high HCV prevalence).

^c^ Excessive alcohol intake was defined as consumption of more than 140 g of pure ethanol per week for women and 210 g of pure ethanol per week for men, corresponding to 14 and 21 glasses of wine, respectively. Present and past consumption were recorded at the moment of the interview.

^d^ The ALT ratio was calculated as function of the upper limit of normal; during the first two periods, this data was already reported as a ratio and during the last period, it was calculated using an upper limit of normal compatible with that used by clinicians during the first period (35 IU/L).

**^e^** Severe liver disease (SLD) was defined by the diagnosis of either cirrhosis or hepatocellular carcinoma.

Overall, a history of intravenous drug use and blood transfusion before 1991 were found in 31.1% and 26.1% of patients respectively.

In 18.7% of all patients, known exposure to a HCV risk factor was what led to HCV infection diagnosis (ranging from 21.6% in 2000–2003 to 7% in 2010–2014) and in 49.5% of cases, it came through systematic screening such as blood donor screening, pre-surgery and pre-transfusion screening, prenatal testing, screening for insurance contract (ranging from 45.2% in 2000–2003 to 73.1% in 2010–2014).

The median time between diagnosis and referral to the expert centre was 4 months (IQR: 2-32) and seemed homogeneous across the three periods, as did the ALT ratio at referral (1.5 times higher than the upper limit of normal) and the proportion of patients with a HBV-co-infection (1.9%). Patients were mainly infected by HCV genotype 1 and 3, with this being in 57.3% and 19.6% of patients with a known genotype, respectively. Of note, the proportion of patients infected with an unknown genotype varied from 35.1% in the first study period to 9.5% in the third.

Of all patients, 10.5% were found to declare a current excessive consumption of alcohol at the time of the interview.

Overall, cirrhosis diagnosis was present at first visit in 10.7% of the patients and HCC was present in 0.9%. Among patients recruited during the third study period, cirrhosis and HCC were present in 25.7% and 2.1% of patients, respectively.

### Factors associated with severe liver disease (SLD)

All selected variables, except for HBV co-infection, were significantly associated with the risk of SLD at patients’ first visit based on the bivariate analysis, and were introduced in the initial regression models. These statistically significant associations were confirmed by the multivariable analysis of imputed data. (**Table 3**)

**Table 3:** Estimation of adjusted prevalence ratios of severe liver disease in patients with chronic hepatitis C at first visit in hepatology expert centres in France using multivariate models in a complete cases analysis and after multiple imputation, France, 2000–2007 and 2010–2014

Patient characteristics		Complete cases multivariate analysis (n = 8,171)					Multiple imputation multivariate analysis (n = 16,851)		
		Number of patients with SLD/total number patients per strata	% SLD^a^ per strata	aPR	95% CI	p value	aPR	95% CI	p value
**Sex**	**Female**	341/3,472	9.8	1	Ref	< 0.001	1	Ref	< 0.001
	**Male**	744/4,699	15.8	1.58	1.40–1.75		1.53	1.40–1.67	
**Country of birth**	**France**	NA	NA	NA	NA	NA	1	Ref	0.002
	**Europe (outside France)**	NA	NA	NA	NA	NA	1.06	0.89–1.26	0.504
	**North Africa and Middle East**	NA	NA	NA	NA	NA	1.34	1.16–1.56	< 0.001
	**Sub-Saharan Africa**	NA	NA	NA	NA	NA	1.00	0.80–1.26	0.975
	**Asia, Pacific, Americas**	NA	NA	NA	NA	NA	0.98	0.78–1.22	0.827
**Age at HCV infection diagnosis**	**18 years**	0/35	0.0	1	Ref	< 0.001	1	Ref	< 0.001
	**19–30 years (25)^b^**	37/1,118	3.3	2.36	2.17–2.57		2.23	2.10–2.38	
	**31–40 years (35)^b^**	124/2,002	6.2	5.69	4.80–6.75		5.09	4.47–5.79	
	**41–50 years (45)^b^**	309/2,175	14.2	10.98	8.68–13.89		9.40	7.87–11.24	
	**51–60 years (55)^b^**	285/1,416	20.1	18.56	13.93–24.72		15.36	12.36–19.09	
	**61–70 years (65)^b^**	196/899	21.8	28.73	20.66–39.94		23.11	18.01–29.67	
	**71–90 years (80)^b^**	133/482	27.6	49.44	33.72–72.50		38.41	28.74–51.33	
**Circumstances of HCV infection diagnosis**	**Systematic screening**	555/4,585	12.1	1	Ref	< 0.001	1	Ref	< 0.001
	**Exposure to a HCV infection risk**	119/1,629	7.3	0.90	0.74–1.08	0.254	0.90	0.78–1.05	0.176
	**Symptoms or laboratory findings**	411/1,957	21.0	1.41	1.26–1.58	< 0.001	1.47	1.34–1.61	< 0.001
**Time between HCV diagnosis and first expert centre visit**	**0 months**	124/672	18.5	1	Ref	< 0.001	1	Ref	< 0.001
	**1–6 months (3)^b^**	496/4,098	12.1	1.02	1.01–1.02		1.01	1.01–1.02	
	**7–12 months (9)^b^**	105/754	13.9	1.05	1.04–1.06		1.04	1.04–1.05	
	**13–24 months (18)^b^**	64/504	12.7	1.11	1.09–1.13		1.09	1.07–1.11	
	**25–48 months (36)^b^**	72/527	13.7	1.22	1.18–1.27		1.19	1.15–1.23	
	**48–96 months (72)^b^**	94/770	12.2	1.50	1.39–1.61		1.42	1.33–1.50	
	**97–192 months (144)^b^**	104/770	13.5	2.24	1.94–2.58		2.01	1.78–2.26	
	**193–288 months (240)^b^**	26/76	34.2	3.83	3.02–4.86		3.19	2.61–3.90	
**French area/region of first visit**	**Paris area**	229/1,466	15.6	1	Ref	0.047	1	Ref	< 0.001
	**North-West**	214/1,833	11.7	0.96	0.81–1.13	0.600	1.25	1.10–1.42	0.001
	**North-East**	190/1,417	13.4	1.00	0.84–1.17	0.955	1.21	1.06–1.39	0.004
	**South-West**	156/1,510	10.3	0.82	0.68–0.98	0.026	1.14	0.99–1.31	0.071
	**South-East**	271/1,777	15.3	1.08	0.93–1.25	0.316	1.38	1.23–1.55	< 0.001
	**French Caribbean islands**	25/168	14.9	1.19	0.83–1.69	0.343	1.53	1.13–2.09	0.007
**Study period at first visit**	**2000–2003** ^c^	358/3,602	9.9	1	Ref	< 0.001	1	Ref	< 0.001
	**2004–2007** ^c^	451/3,697	12.2	1.23	1.08–1.39	0.001	1.31	1.20–1.43	< 0.001
	**2010–2014** ^c^	276/872	31.7	2.14	1.84–2.49	< 0.001	2.39	2.13–2.69	< 0.001
**HCV infection risk factor**	**Intravenous drug use**	282/2,631	10.7	1	Ref	0.038	1	Ref	0.004
	**Nasal drug use**	31/200	15.5	1.21	0.91–1.61	0.196	1.14	0.88–1.48	0.325
	**Blood product transfusion ** **before 1991**	327/2,152	15.2	1.18	0.99–1.40	0.058	1.19	1.04–1.37	0.011
	**Other risk factors**	284/2,104	13.5	1.01	0.86–1.19	0.889	1.00	0.87–1.15	0.967
	**No risk factor found**	161/1,084	14.9	1.25	1.03–1.51	0.026	1.27	1.09–1.47	0.002
**Excessive alcohol intake** ^d^	**None**	624/5,926	10.5	1	Ref	< 0.001	1	Ref	< 0.001
	**Current**	35/257	13.6	1.48	1.08–2.03	0.015	1.39	1.09–1.78	0.009
	**Current and past**	129/637	20.3	2.15	1.81–2.56	< 0.001	2.18	1.90–2.49	< 0.001
	**Past**	297/1,351	22.0	1.90	1.67–2.17	< 0.001	2.08	1.87–2.31	< 0.001
**HCV genotype**	**1**	643/4,701	13.7	1	Ref	< 0.001	1	Ref	< 0.001
	**2**	103/919	11.2	0.81	0.67–0.98	0.027	0.84	0.72–0.98	0.031
	**3**	229/1,602	14.3	1.36	1.19–1.57	< 0.001	1.29	1.15–1.45	< 0.001
	**4**	84/765	11.0	1.07	0.88–1.31	0.487	0.99	0.82–1.19	0.887
	**5, 6 or 7**	26/184	14.1	0.77	0.55–1.08	0.127	0.76	0.57–1.00	0.054
**ALT ratio** ^e^	**1**	51/1,133	4.5	1	Ref	< 0.001	1	Ref	< 0.001
	**1–2.5 (2)^b^**	463/3,684	12.6	1.68	1.52–1.86		1.61	1.50–1.72	
	**2.5–10 (6.5)^b^**	476/1,936	24.6	2.96	2.54–3.45		2.87	2.55–3.22	
	**> 10 (20)^b^**	11/60	18.3	1.40	0.66–2.97		1.80	1.06–3.04	

ALT: alanine aminotransferase; aPR: adjusted prevalence ratio; CI: confidence interval; HCV: hepatitis C virus; NA: not available, not applicable or not known; Ref: reference group for comparison; SLD: severe liver disease.

^a^ Severe liver disease (SLD) was defined by the diagnosis of either cirrhosis or hepatocellular carcinoma.

^b^ Numbers in brackets are the middle value of the category, used to estimate the aPR.

^c^ Data for the time periods 2000–2003 and 2004–2007 came from France’s hepatitis C surveillance network while that for the time period 2010–2014 came from the nationwide multi-centre cohort study ANRS CO22 HEPATHER.

^d^ Excessive alcohol intake was defined as consumption of more than 140 g of pure ethanol per week for women and 210 g of pure ethanol per week for men, corresponding to 14 and 21 glasses of wine, respectively. Present and past consumption were recorded at the moment of the interview.

^e^ The ALT ratio was calculated as function of the upper limit of normal; during the first two periods, this data was already reported as a ratio and during the last period, it was calculated using an upper limit of normal compatible with that used by clinicians during the first period (35 IU/L).

The following factors were associated with an increased risk of SLD at the time of first visit: male sex (aPR = 1.53; 95% CI: 1.40–1.67); being born in North Africa or the Middle East (aPR = 1.34; 95% CI: 1.16–1.56) compared with being born in France; blood product transfusion before 1991 (aPR = 1.19; 95% CI 1.04–1.37) and no other known HCV risk factor (aPR = 1.27; 95% CI: 1.09–1.47) compared with intravenous drug use; current and/or past excessive alcohol intake (p < 0.001); and symptom-based HCV diagnosis compared with diagnosis from systematic screening (aPR = 1.47; 95% CI: 1.34–1.61).

Compared with genotype 1, genotype 2 was found to be negatively associated with SLD while genotype 3 was found to be associated with an increased risk of SLD in both complete case and multiple imputed data analyses.

Age at diagnosis and time between diagnosis and first expert centre visit were linked by a positive nonlinear relationship with the risk of SLD (Table 3). The ALT ratio (cf.d with the upper limit of normal) was linked by a nonlinear non-monotonic relation with the risk of SLD (Table 3).

The prevalence of SLD significantly changed across the three study periods (p < 0.001 in both analyses) and doubled between the first period (2000–2003) and the last (2010–2014).

Apart from the country of birth, which was not significantly associated with the risk of SLD in the complete cases analysis, results from the complete cases multivariate analysis (n = 8,171) and multiple imputation multivariate analysis (n = 16, 851) were similar.

We found no statistically significant interaction.

## Discussion

Our observational multi-centre study allows us to describe factors associated with SLD in patients with chronic hepatitis C at the time of their first visit to a hepatology expert centre in France from 2000 to 2007 and 2010 to 2014. It confirms the influence of several known risk factors for SLD in chronic hepatitis C, including male sex, a history of excessive alcohol intake, age at diagnosis and HCV genotype 3 [6,11,21].

During the overall study period we observed a general increase in the percentage of SLD at first visits. This trend is consistent with those observed via the French Hospital Discharge Data System (PMSI) from 2004 to 2011, where both diagnosis of cirrhosis and of HCC among the HCV-infected hospitalised population increased from 17.8% to 33.7% and from 4.0% to 7.3%, respectively [22]. During the same period, the prevalence of chronic hepatitis C tended to decrease in the hospitalised population (from 0.45% to 0.33%) and general population (from 0.53% to 0.42%) in France [22]. These opposing trends could reflect an ageing of patients infected with HCV in recent decades and fewer new HCV infections in France. Patients’ evaluation at an expert centre was a key moment before specific treatment, and in general, patients with SLD may have been referred for follow-up and treatment more frequently than non-severe patients. During the 2012–2014 period, which corresponds with the start of HCV treatment with DAAs, patients with severe liver fibrosis or SLD had priority access to these innovative treatments. In this context, patients with SLD were probably referred to expert centres for treatment by their general practitioner more frequently than in the past. It may therefore be the case that the evolution observed in our data reflects increased attractiveness of specialised care services during the last period. In addition, it is possible that patients with SLD were more likely to have been included in the ANRS CO22 HEPATHER cohort by the expert centres themselves as one of the cohort’s objectives is to evaluate and measure the impact of new drug associations on the course of chronic hepatitis C. Consequently, it is possible that the burden of SLD we observed in our study is overestimated, especially for the third period of study, although the change in SLD prevalence might be consistent with an existing trend in French chronic hepatitis C epidemiology.

We found that the longer the time between HCV infection diagnosis and first hepatology expert centre visit, the higher the probability was of having SLD. This highlights the urgent need to raise the awareness among patients and general practitioners about the need for both close monitoring of chronic HCV infection after diagnosis and earlier referral for treatment.

Interestingly, our results suggest a protective effect of genotype 2 on the risk of SLD at a patient’s first expert centre visit. Indeed, there is already some evidence linking genotype 2 to a lower prevalence of liver fibrosis [23] and to slower progression to cirrhosis [24,25] compared with other genotypes. We also confirmed the relationship between genotype 3 and liver cirrhosis shown by others [21,25].

Place of birth appears to be linked to the risk of SLD at first hepatology expert centre visit, especially for patients born in North Africa or the Middle East. Although an association between migration and poorer prognosis in hepatitis C has already been described [26,27], it is not well understood and needs further investigation. One possible reason for this observed association is the lack of information concerning comorbidities such as type 2 diabetes and metabolic syndrome, which are potentially not homogeneously prevalent among different ethnic groups. In addition, the evolution of chronic hepatitis C in this sub-group of patients could be different. Age at infection, mode of contamination, lifestyle, access to HCV screening and care may all influence the course of disease progression [28,29]. Another possible reason for the association between place of birth (as a proxy for patients’ country) and the risk of SLD at first hepatology expert centre visit could be that people from France might be referred to an expert centre even when not severely ill, while patients from foreign countries (especially those that are mainly francophone) might more frequently seek treatment at an expert centre in France only when they become severely ill.

Age at diagnosis had a positive relationship with the risk of SLD in our study. This variable takes the age of the patient, a known risk factor for the evolution to cirrhosis [30], into account. Age at diagnosis may also reflect the duration of infection: the older the patient at diagnosis, the more likely he or she has been infected longer. The importance of early diagnosis is indicated by the increased risk of SLD at the time of first expert centre visit for patients whose diagnosis is based on hepatic or digestive symptoms (clinical or biochemical).

In addition, our study showed that patients with no identified HCV infection risk factor tended to have a greater probability of SLD at their first visit than well-identified at-risk groups, such as drug users and blood transfusion recipients before 1991. This finding suggests that when both patients and practitioners are unaware of the risk of viral hepatitis, the former tend to be referred when they already have late stage disease and a poorer prognosis.

The limitations of our study include: (i) its cross-sectional design, with retrospective and self-reported assessment of several exposures, including alcohol consumption and risk behaviours, (ii) the inclusion of one group of patients via systematic surveillance and the other via a cohort study, with different recruitment and with slight differences in the forms’ wording and structure, that has probably lead to a difference in the quality of data and in missing data proportions, and (iii) other factors known to be associated with fibrosis (e.g. obesity, metabolic syndrome, type 2 diabetes, duration of infection) were not collected for all the periods, with these factors therefore not included in our analysis. Furthermore, our data could only provide estimations on a select population (chronic hepatitis C patients referred to a hepatology expert centre), not necessarily reflecting the characteristics of the overall population of patients with chronic hepatitis C.

Despite these study limitations, our work provides interesting insights in the context of chronic hepatitis C patient care evolution.

First, it underlines the importance of early diagnosis and providing the general population with better information about HCV infection risk factors. In fact, severe disease was more frequently diagnosed when symptoms or biochemical liver abnormalities triggered testing. To increase people’s awareness about their HCV infection status, screening recommendations and guidelines were revised [31,32] to emphasise both targeted and mass screening. Accordingly, the utilisation of HCV rapid tests has been authorised in the context of healthcare facilities and approved charities.

Second, our results highlight that a long delay between diagnosis and first visit to a hepatology expert centre increases the risk of having late stage disease when starting specialised care and antiviral treatment. This in turn is associated with a lower probability of treatment success and the continued risk of further complications even after successful treatment. In this light, the French National Authority for Health (HAS) issued new recommendations for treatment in June and December 2016, broadening the eligibility parameters for DAAs treatment to include patients with a METAVIR score of F0-F1, incorporating both individual and collective objectives for HCV infection eradication [32,33], and allowing the prescription of DAAs outside the expert centres. Health authorities should also urge general practitioners to refer patients with HCV infections to a hepatology specialist or unit as early as possible in order to ensure their steady treatment. As now recommended in France, all adults diagnosed with hepatitis C should be immediately referred for evaluation and HCV treatment.

Finally, particular attention should be paid to migrant patient as they could be at greater risk of already having late stage disease when referred to specialised care. Early referral is even more important for patients with supplementary risk factors such as a history of alcohol excessive consumption, HCV infection diagnosis at an older age, male sex and HCV genotype 3.

These findings originating from French epidemiological data may be important for other European countries dealing with similar challenges in HCV infection care. Indeed, effective tertiary prevention of HCV complications, which have been made possible thanks to the extension of DAAs treatment to new populations, is an ethical obligation of our modern healthcare systems. In this light, effective large-scale screening and earlier referral of HCV-infected patients are two very important public health tools.
